# PanSNPdb: The Pan-Asian SNP Genotyping Database

**DOI:** 10.1371/journal.pone.0021451

**Published:** 2011-06-23

**Authors:** Chumpol Ngamphiw, Anunchai Assawamakin, Shuhua Xu, Philip J. Shaw, Jin Ok Yang, Ho Ghang, Jong Bhak, Edison Liu, Sissades Tongsima, and the HUGO Pan-Asian SNP Consortium

**Affiliations:** 1 National Center for Genetic Engineering and Biotechnology (BIOTEC), Klong Luang, Pathumthani, Thailand; 2 Inter-Department Program of BioMedical Sciences, Faculty of Graduate School, Chulalongkorn University, Bangkok, Thailand; 3 Chinese Academy of Sciences and Max Planck Society (CAS-MPG) Partner Institute for Computational Biology, Shanghai Institutes for Biological Sciences, Chinese Academy of Sciences, Shanghai, China; 4 Korean BioInformation Center (KOBIC), Korea Research Institute of Bioscience and Biotechnology (KRIBB), Yuseong-gu, Deajeon, South Korea; 5 Personal Genomics Institute, Genome Research Foundation, Suwon, South Korea; 6 Theragen BiO Institute, TheragenEtex, Suwon, South Korea; 7 Genome Institute of Singapore, Singapore, Singapore; Ohio State University Medical Center, United States of America

## Abstract

The HUGO Pan-Asian SNP consortium conducted the largest survey to date of human genetic diversity among Asians by sampling 1,719 unrelated individuals among 71 populations from China, India, Indonesia, Japan, Malaysia, the Philippines, Singapore, South Korea, Taiwan, and Thailand. We have constructed a database (PanSNPdb), which contains these data and various new analyses of them. PanSNPdb is a research resource in the analysis of the population structure of Asian peoples, including linkage disequilibrium patterns, haplotype distributions, and copy number variations. Furthermore, PanSNPdb provides an interactive comparison with other SNP and CNV databases, including HapMap3, JSNP, dbSNP and DGV and thus provides a comprehensive resource of human genetic diversity. The information is accessible via a widely accepted graphical interface used in many genetic variation databases. Unrestricted access to PanSNPdb and any associated files is available at: http://www4a.biotec.or.th/PASNP.

## Introduction

In recent years, genome-wide single nucleotide polymorphism (SNP) data from high density array platforms and next generation whole-genome sequencing data have been gathered from various human populations. These data embody the transition from single-locus based studies to genomics analyses of human population structure and disease gene mapping [1]–[5]. Until recently, Asian populations have been largely underrepresented in genome-wide studies in comparison to other peoples of the world. For example, both the International HapMap project and 1000 Genome project lack population samples from Southeast Asia, which is known to contain the most ethno-linguistically diverse populations in Asia. To address this type of shortcoming, the Human Genome Organization (HUGO) Pan-Asian SNP consortium was established to sample genetic diversity in Asia. This effort culminated in a survey of 1,719 unrelated individuals from 71 populations from China (including Taiwan), India, Indonesia, Japan, Malaysia, the Philippines, Singapore, South Korea and Thailand [6]. These 71 populations represent most of the major linguistic groups in Asia and the Pacific, i.e. Altaic, Austro-Asiatic, Austronesian, Dravidian, Hmong-Mien, Indo-European, Papuan, Sino-Tibetan and Thai-Kadai. Considering the general concordance between linguistic and genetic affiliations of human populations, genome-wide data from these samples also captured the majority of the human genetic diversity in Asia. A distinct north - south cline with increasing genetic diversity was observed and contrary to the two-wave migration hypothesis, our study showed substantial genetic proximity of Southeast Asian and East Asian populations [6]. This suggested that the entry of humans into the Asian continent occurred as a single primary wave, populating the south and then expanding northward.

Beside population genetics, there are many other uses of this information include pharmacogenomics, forensics, and genetic epidemiology. The complexity of this dataset poses difficulties for analysis, since only the genotypic transformations of the data are available from the SNP database from National Center for Biotechnology Information (dbSNP), and are thus accessible only to researchers with advanced bioinformatic capabilities. Hence, a database of various analyses accompanying the data would be of benefit to researchers in different disciplines who may not have the bioinformatic capabilities to obtain the information they require.

The goals of the Pan-Asian SNP database are 1) present the data in different formats to facilitate analysis with different tools by providing a graphical viewing interface; 2) comparison of the Pan-Asian dataset with other genetic variation databases including HapMap3 [7], dbSNP [8], and Japan SNP database (JSNP) [9]; 3) incorporate the results of different analyses, including the previously published patterns of population genetic structure and new analyses (linkage disequilibrium patterns, haplotype blocks inferred from the linkage disequilibrium (LD) patterns, tagSNPs as markers of LD blocks, copy number variations (CNVs) inferred from the SNP raw data); and 4) provide an infrastructure for future deposition of data and analysis pertaining to Asia.

## Results and Discussion

### Genotyping and allele frequencies

Genotyping of Affymetrix GeneChip Human Mapping 50K Xba arrays was performed at eight different genotyping centers (China, India, Japan, Korea, Malaysia, Singapore, Taiwan and USA), according to the manufacturer's protocols. More information regarding SNP calling can be found in the Supplements of [6]. In addition to these HUGO Pan-Asian SNP consortium data, the data for the matching SNPs from 209 HapMap samples (CEU, CHB, JPT and YRI) were included into PanSNP. The final dataset contained the genotypes of 54,794 and 1,204 SNPs mapping to autosomal and sex chromosomes respectively for each individual.

### Haplotype inference and block partitioning

Haplotype blocks were predicted exclusively on autosomal chromosomes using HaploBlockFinder [10] using 1928 individuals from 75 populations (excluding AX–AI) based on the four gamete test (FGT) assumption with parameters:

–A3 –D0.8 –B0.01 –M1 –T1 –P0.8 –Q0.2

The haplotypes of each block were inferred using fastPHASE [11] with parameters:

–T20 –C50 –Km1000 –Kp.05

**Figure 1:**
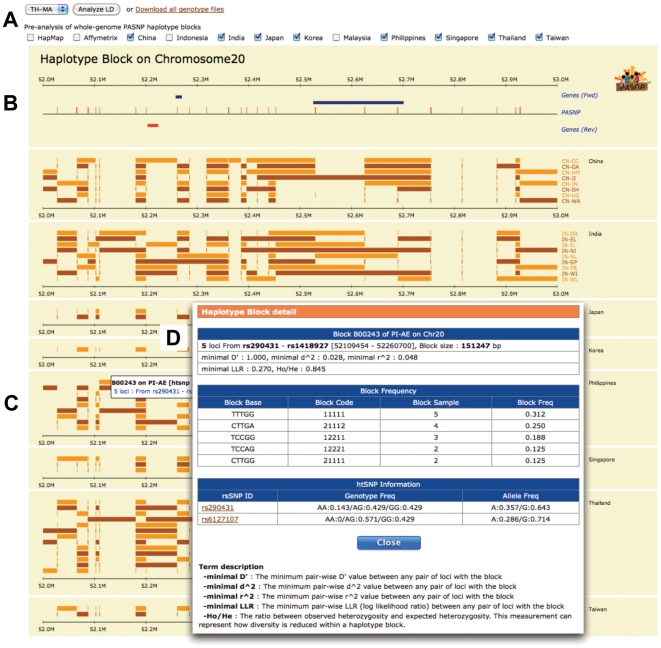
Representation of Haplotype blocks A) haplotype blocks calculation and population selection panel B) SNPs and genes located on chromosome 20 between 52–53 Mb displayed in SVG C) haplotype blocks of the selected populations and D) detailed information (block frequency, tag SNPs) of haplotype blocks displayed by clicking on the SVG view.

The blocks and their haplotypes are stored in the database and can be graphically displayed through the web interface shown in Figure 1. Detail on SNP distribution of each chromosome is listed in Table S1.

### Copy number variation analysis

**Figure 2:**
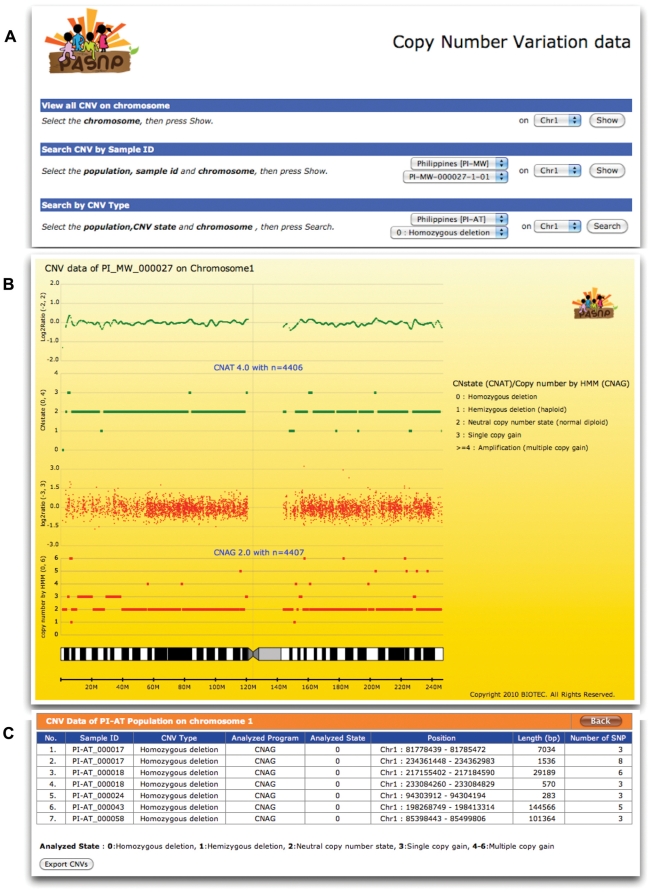
Copy Number Variation view A) interface to view CNV information B) CNV data of each individuals in SVG, showing log2ratio of signal intensity plots and called states from CNAT 4.0 and CNAG 2.0 programs C) individual CNV results on each chromosome corresponding with CNV type selected in panel A.

Copy number analyses were done using Copy Number Analysis Tools version 4.0 (CNAT4.0) [12] and Copy number analyzer for GeneChip(CNAG 2.0) [13]. Since the focus is on the population level, un-paired sample analysis with 1 Mbps genomic smoothing was used in these analyses. Male and female data were analyzed separately for chromosome X. The CNV graphical interface shown in Figure 2 displays the log_2_ratio of the probe intensities and CNstate/N_AB results from CNAT4.0 and CNAG2.0 respectively. More information on CNV analysis can be found in Text S2.

### Conclusion

Following the publication of the HUGO Pan-Asian SNP consortium study of human genetic diversity in Asia, it became apparent that there was a need for an information resource which integrates the Asian data with other worldwide populations and presents this data is a user friendly format. Similar to the HapMap initiative, PanSNPdb offers genome structural information pertaining to Asian populations in a familiar graphical comparative view based on GBrowse where SNP genotyping from multiple populations can be visualized on the same page. This database also offers pre-computed information of LD blocks and their haplotypes on each chromosome; such information for each population can be visualized both in table and SVG formats and can be exported for future use. Furthermore, users can adjust the number of SNPs for haplotype inferencing and calculate this using Haploview, which is performed by our server. In terms of genome structure, we calculated the CNV information using un-paired sample analysis whose information, e.g., log2ratio and CNV state for individual visualization (SVG) and CNV state at the population level (GBrowse) comparing with CNV information from the database of genomic variations. The database is available for public access at: http://www4a.biotec.or.th/PASNP.

This database offers a comprehensive catalog of Asian population genotypes, which is compatible with the HapMap project. It also serves as the main genotyping repository of the Pan Asian SNP consortium which will contribute further Asian specific genetic information in the future. We anticipate that newer Asian populations with denser genotyping platform along with their analyses from the consortium will be deposited into PanSNPdb. With the advent of more cost effective whole genome sequencing technology, other structural genomic variations among Asian populations will also be explored.

## Methods

### System Design and Implementation

PanSNPdb manages the genetic variation data, reference information and precomputed haplotypes using the open source database management MySQL version 5.5.1. The web interface was constructed using the content management system (CMS) Plone version 3.3.5. Python scripting language was used to connect to MySQL and draw scalable vector graphic (SVG) images of precomputed haplotype blocks and CNV log2ratio signals. PanSNPdb adopts GBrowse to display population-level comparison of SNP and CNV locations on genes and chromosomes. The database system is hosted by a dedicated computer server equipped with 2xAMD 6-core with a clock speed of 2.8 GHz using 64 Gigabytes of memory and 2 Terabytes of hard disk space.

**Figure 3:**
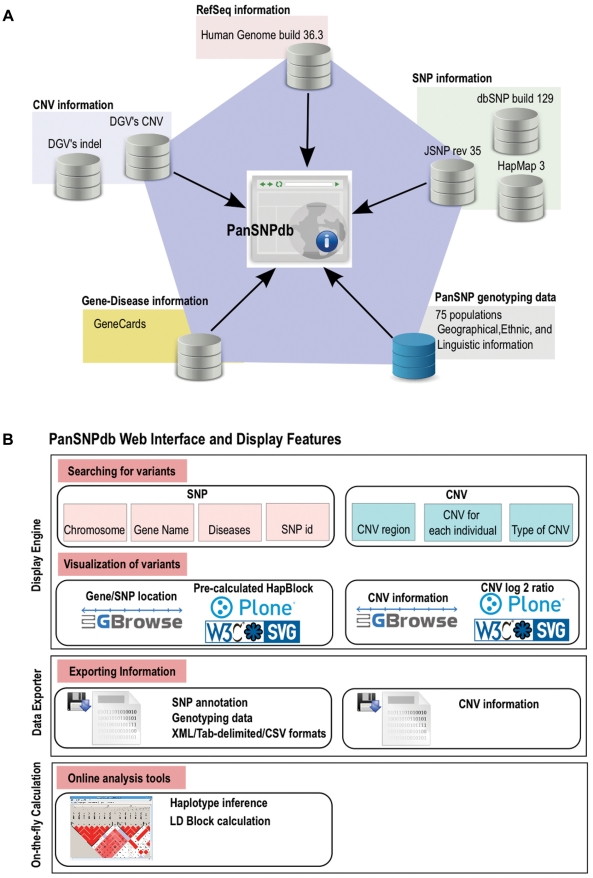
Structure of PanSNPdb A) Architecture of PanSNPdb showing integration of different data sources B) PanSNPdb Web interface and display features.

The PanSNPdb database was constructed using the genotyping information described in [6] consisting of 1,928 unrelated individuals representing 71 Asian populations and 4 populations from HapMap. Information related to each population, such as geographical, ethnic and linguistic data were added to the database; this information is provided in Table S2 and can be visualized through the PanSNPdb web interface. The database was designed and implemented so as to facilitate comparison with genotyping information from other public data sources including HapMap, dbSNP and JSNP. To locate SNPs, the Reference Sequence of Human Genome build 36.3 is used as the template. Since these SNPs may be useful for medical genetic studies, the gene-disease information published by GeneCards was incorporated into the database. These reference data were downloaded, and will be periodically updated when newer versions are announced. Furthermore, copy number variations from the PanAsian SNP dataset were inferred using CNAT and CNAG for future CNV referencing of Asian populations. CNV data from the database of genomic variants (DGV) [14] were incorporated into PanSNPdb so that the comparative view of CNVs across different populations can be rendered. Figure 3A presents the main data sources of the PanSNPdb. Consequently, the comprehensive information in this PanSNPdb can be considered as worldwide data collection, but with special emphasis on Asian populations.

### Graphical interface of the data

Figure 3B shows how the graphical interface of PanSNPdb was constructed. In PanSNPdb, SNPs and their corresponding information can be located graphically on the reference sequence along with SNPs from other populations in different tracks. This visualization is made possible using the GBrowse visualization engine [15]. SNPs can be searched via four main entry points: 1) chromosomal location 2) gene name/gene id 3) SNP id or rs number and for medical purpose 4) disease name from GeneCards that are associated with disease-related genes. Similarly, the CNV region information can also be visualized using GBrowse along with other CNVs from DGV.

**Figure 4:**
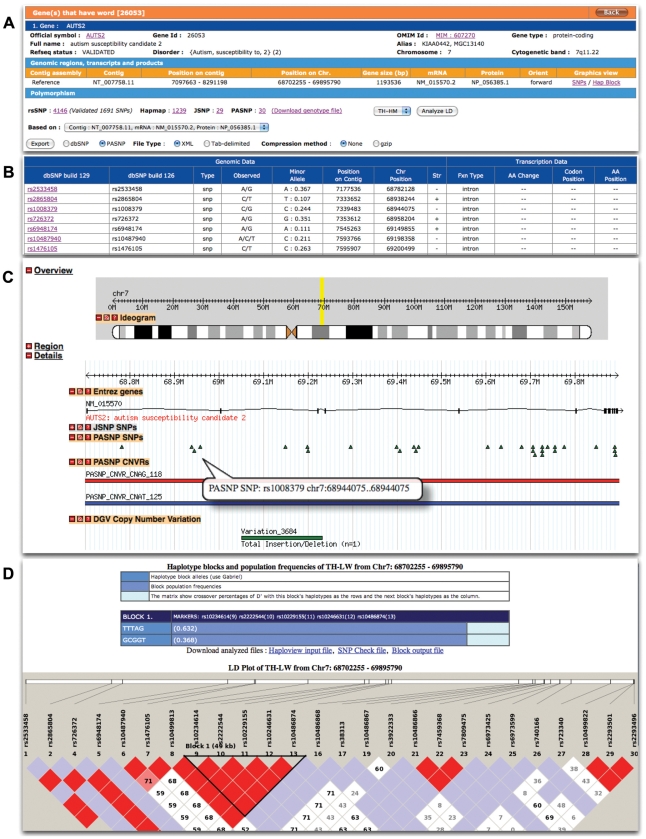
PanSNP results in rich text and graphical formats A) gene and SNPs information that provide export and analyze features B) SNPs and associate informations C) SNPs and genes display with GBrowse D) haplotype blocks calculation with built-in haploview.

Haplotype blocks were also inferred at the chromosome level (autosomes) with overlapping regions (see Table S1 for distribution of SNPs on each chromosomes). The results can be displayed graphically in any web browser with scalable vector graphic (SVG) supported. The Haploview tool [16] is also integrated into the PanSNPdb website; users can adjust the haplotype inferencing parameters in order to recalculate haplotype blocks “on-the-fly”. Lastly, PanSNPdb allows users to export SNP and CNV data, such as location of SNPs, genotyping and CNV data of each individual (in comma separated value (CSV) and/or tab delimited formats). Figure 4 show representative SNP data with beautified text format and a user-interactive graphical view.

## Supporting Information

Text S1 The participants of the HUGO Pan-Asian SNP Consortium are arranged by surname alphabetically.
(DOC)

Text S2 PanSNPdb CNV analysis.
(PDF)

Table S1 Total number of SNPs and CNVs (map on RefSeq Genome Build 36.3).
(XLS)

Table S2 Information of 71 Pan-Asian and 4 HapMap populations.
(XLS)
