# Supplementary material for: PanSNPdb: The Pan-Asian SNP Genotyping Database
Source: PLoS One. 2011 Jun 23;6(6):e21451. doi: 10.1371/journal.pone.0021451 (PMC3121791; doi:10.1371/journal.pone.0021451)
Supplement: Text S2 — PanSNPdb CNV analysis. (PDF) [file pone.0021451.s002.pdf]

PanSNPdb CNV analysis

CNAT 4.0

[http://media.affymetrix.com:80/support/technical/whitepapers/cnat_4_algorithm_whitepaper.pdf]

1. Running Conditions:
   A. Un-paired Sample Analysis
   B. 1 Mb Genomic Smoothing
   C. Run Male/Female (ChrX) separately
2. Filtering Conditions:
   A. Two samples (MY-BD-000032-1-01, SG-CH-000018-1-01) have errors in their CEL files as raw data.
   B. Exclude samples having genotype Call Rate < 93% (based on BRLMM algorithm)
   C. Exclude 46 no mapping genomic mapping Probe sets (based on build 35)
3. Results

   55,979 of 56,025 probes' log2ratio and CN states for the 1633 individuals
   1. log2ratio using smoothed log2ratio value
   2. CN states using HMM copy number state

      A 5-State Hidden Markov Model (HMM) is applied for smoothing and segmenting the CN data
      1. CN State = 0 : Homozygous deletion
      2. CN State = 1 : Hemizygous deletion (haploid)
      3. CN State = 2 : Copy neutral (normal diploid)
      4. CN State = 3 : Single copy gain
      5. CN State >= 4 : Amplification (multiple copy gain)

CNAG 2.0

[http://www.genome.umin.jp/CNAGdownload2.html]

1. Running Conditions:
   A. Non-paired Sample Analysis
   B. Run Male/Female (ChrX) separately
2. Filtering Conditions:
   A. Two samples (MY-BD-000032-1-01, SG-CH-000018-1-01) have errors in their CEL files as raw data.
3. Result

   56,026 probes' log2ratio and N_AB values for the 1742 individuals
   1. log2ratio_AB is log2ratio of signal intensity
   2. `log2((signal_A_sample+signal_B_sample)/(signal_A_reference+signal_B_reference))`
   3. N_AB is copy number estimated by Hidden Markov mode (HMM)
   4. Missing values: There are some error values (-1.#INF00) in 7 probe sets (SNP_A-1728187, SNP_A-1647122, SNP_A-1752709, SNP_A-1716781, SNP_A-1702169, SNP_A-1685724, and SNP_A-1661203)
